# Supplementary material for: Discovery of a proteolytic flagellin family in diverse bacterial phyla that assembles enzymatically active flagella
Source: Nat Commun. 2017 Sep 12;8:521. doi: 10.1038/s41467-017-00599-0 (PMC5595980; doi:10.1038/s41467-017-00599-0)
Supplement: Supplementary file 1 — Supplementary Information [file 41467_2017_599_MOESM1_ESM.pdf]

Description of Supplementary Files

File name: Supplementary Information
Description: Supplementary figures, supplementary tables and supplementary discussion.

File name: Peer review file

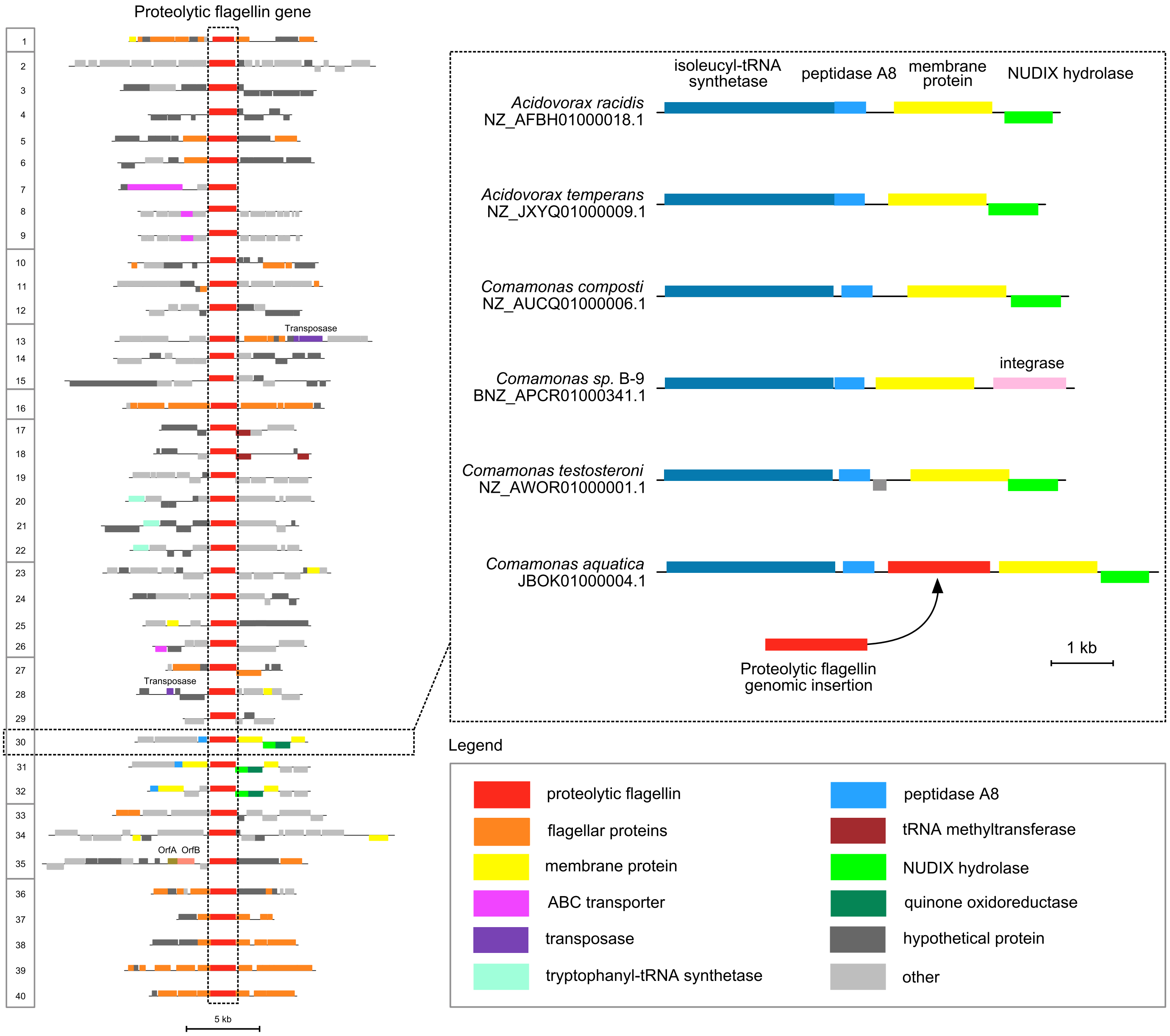

Supplementary Figure 1 | Genomic context surrounding the proteolytic flagellin gene in 40 genomes (left) and evidence for a proteolytic flagellin gene insertion into a conserved *Comamonadaceae* gene cluster (right). The 40 gene clusters are ordered into ten clusters based on a phylogeny of the flagellin protease domain. Species and NCBI accession numbers corresponding to the numbering in the left panel is as follows: 1 - *Lysinibacillus sinduriensis* BLB-1 JPVO01000045.1; 2 - *Eubacterium cellulosolvens* ATCC 43171 NZ_CM001487.1; 3 - *Lachnospiraceae* bacterium M18-1 NZ_KE159611.1; 4 - *Dorea* sp. 5-2 NZ_KE159737.1; 5 - *Butyrivibrio* sp. LC3010 NZ_AUJU01000005.1; 6 - *Butyrivibrio* sp. AC2005 NZ_AUJI01000009.1; 7 - *Pseudobutyrivibrio* sp. MD2005 NZ_JHXE01000016.1; 8 - *Butyrivibrio* sp. AD3002 NZ_ATVV01000009.1; 9 - *Butyrivibrio* sp. WCD2001 NZ_KE384200.1; 10 - *Colwellia piezophila* ATCC BAA-637 NZ_KB905162.1; 11 - *Pseudoalteromonas tunicata* D2 NZ_AAOH01000002.1; 12 - *Ferrimonas kyonanensis* DSM 18153 NZ_KE384363.1; 13 - *Desulfitobacterium dichloroeliminans* LMG P-21439 NC_019903.1; 14 - *Oceanospirillum beijerinckii* DSM 7166 NZ_AULT01000024.1; 15 - *Oceanspirillum* maris DSM 6286 NZ_KE383937.1; 16 - *Desulfuromonas acetoxidans* DSM 684 NZ_AAEW02000008.1; 17 - *Psychromonas ossibalaenae* ATCC BAA-1528 NZ_KB906968.1; 18 - *Psychromonas aquimarina* str. ATCC BAA-1526 NZ_AUAM01000014.1; 19 - *Azoarcus* sp. BH72 NC_008702.1; 20 - *Massilia* sp. JS1662 JPQD01000035.1; 21 - *Massilia niastensis* DSM 21313 NZ_KB908111.1; 22 - *Massilia alkalitolerans* DSM 17462 NZ_ATYR01000022.1; 23 - *Desulfosporosinus orientis* DSM 765 NC_016584.1; 24 - *Pseudomonas alcaligenes* OT 69 NZ_ATCP01000169.1; 25 - *Methyloversatilis* sp. NVD NZ_KB889967.1; 26 - *Chitinibacter tainanensis* DSM 15459 NZ_AUCN01000004.1; 27 - *Bacillus firmus* DS1 APVL01000005.1; 28 - *Aquaspirillum serpens* DSM 68 NZ_ATVC01000046.1; 29 - *Acidovorax* sp. CF316 NZ_AKJX01000208.1; 30 - *Comamonas aquatica* DA1877 JBOK01000004.1; 31 - *Hylemonella gracilis* ATCC 19624 NZ_AEGR01000102.1; 32 - *Hylemonella gracilis* str. Niagara R JEMG01000001.1; 33 - *Clostridium saccharoperbutylacetonicum* ATCC 27021 NC_020291.1; 34 - *Acetobacterium woodii* ATCC 29683 NC_016894.1; 35 - *Selenomonas ruminantium* subsp. lactilytica str. TAM 6421 NC_017068.1; 36 - *Paenibacillus wynnii* str. DSM 18334 JQCR01000002.1; 37 - *Clostridium novyi* A str. BKT29909 JENM01000026.1; 38 - *Clostridium novyi* A str. 4570 JDRX01000001.1; 39 - *Clostridium botulinum* C/D str. DC5 JDRY01000001.1; 40 - *Clostridium haemolyticum* NCTC 9693 JENX01000076.1

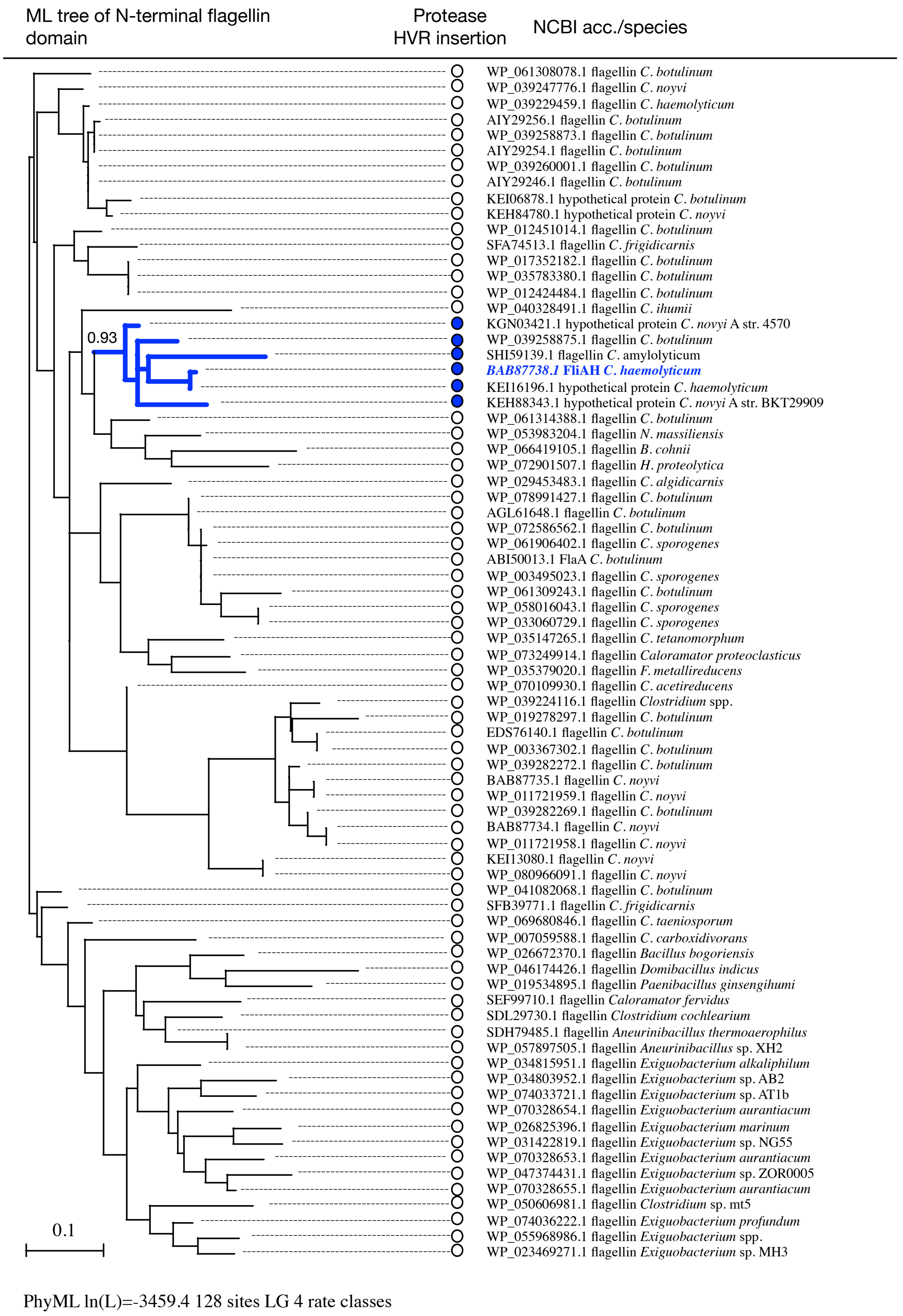

Supplementary Figure 2 | Phylogeny of the top BLAST hits to the N-terminal flagellin domain of *C. haemolyticum* FliA(H) (flagellinolysin, NCBI accession number BAB87738.1). Flagellinolysin forms a well-supported (0.93 aLRT value) monophyletic cluster with related sequences that contain a unique protease domain insertion within their hypervariable region. The protease domain is absent in all other members of this tree.

LC-MS/MS analysis of Trx::FliA(H)-HVR by trypsin

92% sequence coverage

```
  1 MSDKIIHLTD DSFDTDVLKA DGAILVDFWA EWCGPCKMIA PILDEIADEY
 51 QGKLTVAKLN IDQNPGTAPK YGIRGIPTLL LFKNGEVAAT KVGALSKGQL
101 KEFLDANLAG SGSGHMHHHH HHSSGLVPRG SGMKETAAAK FERQHMDSPD
151 LGTDDDDKMK GLKTGWIEKS VENIKTAYGI EPTGANKLKV TISDDGAYGV
201 LASVTPKTGE FELHIDSSDF EKGDGESGNN IHGKLYDDRI IQHEMTHAVM
251 NDALGIDKMN DLHDKNKLWF IEGTAEAMAG ADERVKDIIG NDTQTGIDNT
301 KLSKLATRAD ALLNGVSWNS SDEDYAAGYL MVKYIASKGI DLKAVMKEIK
351 NTGASGLDNK IDLTNLKIDF KNNLENYIKD ISKVHLDWDD DEKDVGSILG
401 SDHGHGDIKA EDVVKGTTPE KEQPLDKFKI IWPDDNSDNT TGKIQLQVGA
451 NEGQSITILE
```

Blue: trx-domain; Green: hexahistidine-tag; Red: enterokinase cleavage site; Black: peptidase domain of *C. haemolyticum* FliA(H). **Peptides in bold were identified by MS/MS analysis at a false discovery rate (FDR) of 1%.**

Supplementary Figure 3 | LC-MS/MS results for the recombinant FliA(H)-HVR protein. Sequence coverage of FliA(H)-hypervariable region (HVR) based on peptides identified by LC-MS/MS at an FDR ≤ 1%. Trx, thioredoxin-tag on the recombinant protein.

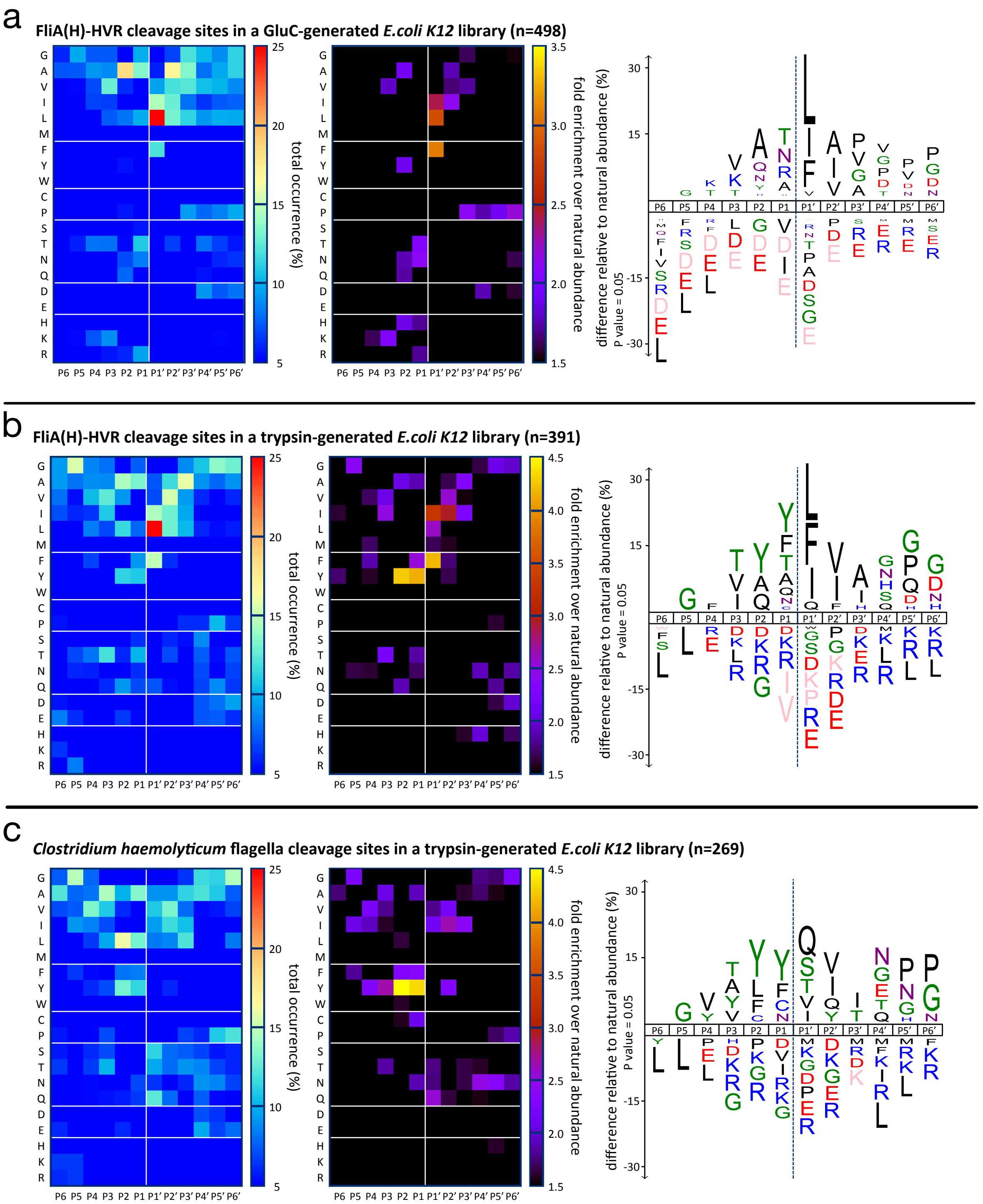

Supplementary Figure 4 | PICS MS/MS assay results for recombinant *Clostridium haemolyticum* FliA(H)-hypervariable region (flagellinolysin) and purified flagellar sheared filaments. Cleaved peptide sequences identified from PICS MS/MS assays presented as heat maps and iceLogos for the recombinant FliA(H)-hypervariable region (HVR) protein from *Clostridium haemolyticum* using *E. coli* K12 GluC-prepared (**a**) and trypsin-prepared (**b**) peptide libraries. 498 and 391 cleavage sites were determined and a sequence logo derived, respectively. (**c**) PICS MS/MS assay results for purified flagellar filaments sheared from *C. haemolyticum* using a trypsin-prepared peptide library, which identified 269 cleavage sites.

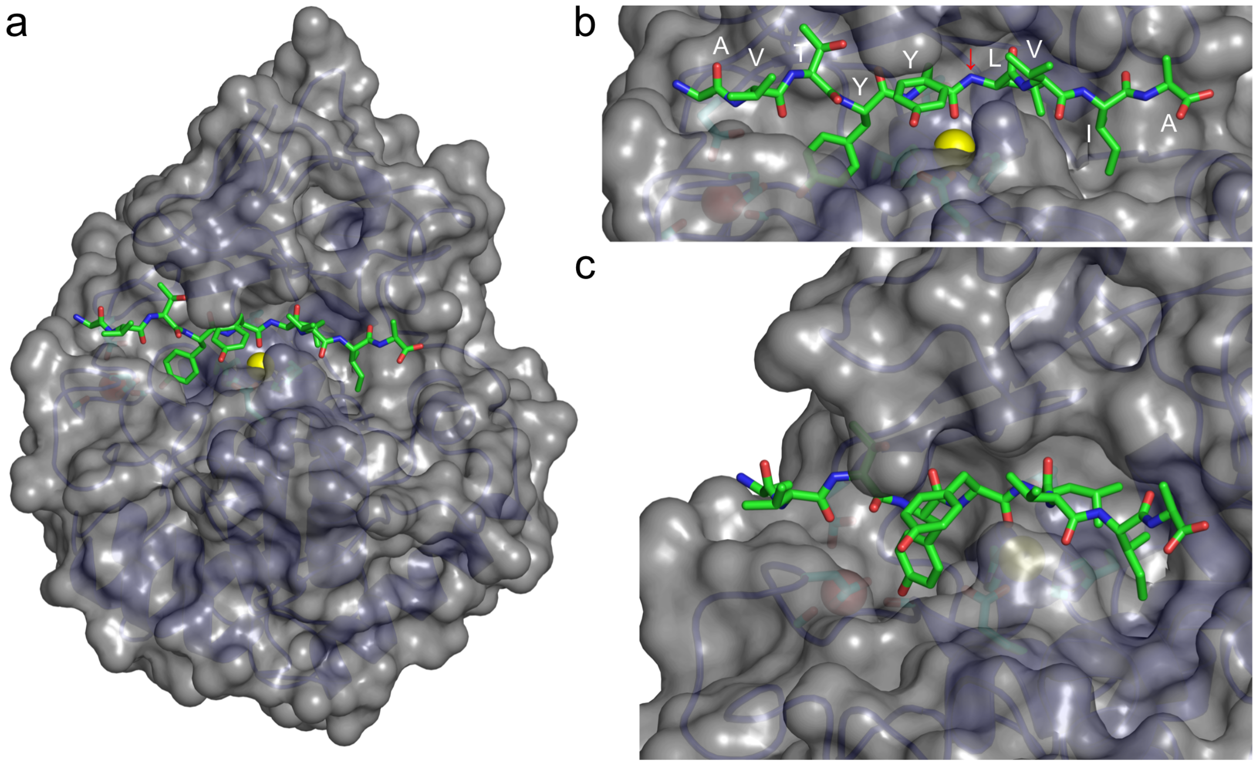

Supplementary Figure 5 | Peptide docking model using the predicted structure of the protease domain of *Clostridium haemolyticum* FliA(H) and the (AVTYY ↓ LVIA) nonapeptide based on the consensus specificity determined by PICS. (**a**) Whole view of FliA(H) protease domain-peptide docking model. (**b**) A zoomed view of the peptide-binding groove with peptide residues labeled in white and the cleavage site indicated by a red arrow. (**c**) A second zoomed view rotated 25 degrees counterclockwise revealing steric complementarity between the peptide and binding groove.

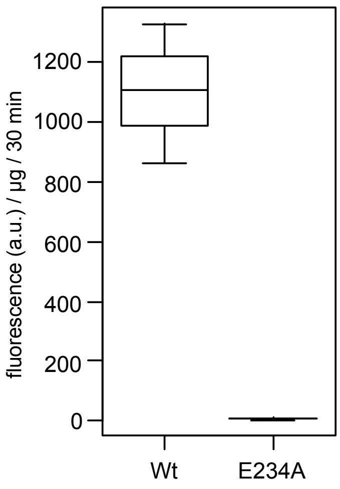

Supplementary Figure 6 | Fluorometric peptidase assays for FliA(H)-HVR (wild type) *versus* inactive mutant FliA(H)-HVR (E234A) using the quenched fluorescent peptidic substrate with the sequence ALGL. A box plot is shown with the activity normalized over three time points and calculated from total of six data points per sample. Box plots show the minimum value (bottom line), lower quartile (bottom line of box), median value (middle line in box), upper quartile (top line of box), and maximum value (top line). Box plots were created using the boxplot() function in R.

tr|A0A0A0IT63|A0A0A0IT63_CLOHA (100%), 61,664.0 Da
Flagellin OS=Clostridium haemolyticum NCTC 8350 GN=fliA(H) PE=3 SV=1
37 exclusive unique peptides, 68 exclusive unique spectra, 136 total spectra, 442/561 amino acids (79% coverage)

MIINHNINAM	NAHRRIGINT	VKSGKAMEKL	SSGLRINRAG	DDAAGLAISE	KMRGQIRGLN
QASRNAQDGI	SLIQTAEGAL	NETHAIVQRM	RELSVQAAND	TNTKDDREQI	QKEIKQLIEE
VDRIGNTTEF	NTIKLLTSGV	KPEVKENIIK	GLKTGWIEKS	VENIKTAYGI	EPTGANKLKV
TISDDGAYGV	LASVTPKTGE	FELHIDSSDF	EKGDGESGNN	IHGKLYDDRI	IQHEMTHAVM
NDALGIDKMN	DLHDKNKLWF	IEGTAEAMAG	ADERVKDIIG	NDTQTGIDNT	KLSKLATRAD
ALLNGVSWNS	SDEDYAAGYL	MVKYIASKGI	DLKAVMKEIK	NTGASGLDNK	IDLTNLKIDF
KNNLENYIKD	ISKVHLDWDD	DEKDVGSILG	SDHGHGDIKA	EDVVKGTTPE	KEQPLDKFKI
IWPDDNSDNT	TGKIQLQVGA	NEGQSITISL	KDMRSSALGI	EKLEVTSFES	ASQSIKSCDN
AIEKISNFRS	ELGAYQNRLE	HTINNLNTSA	ENLQASESRI	RDVDMAKEMM	NFSKNNILAQ
AAQAMLAQAN	QQPQGILQLL	R			

Supplementary Figure 7 | Sequence coverage of the *Clostridium haemolyticum* FliA(H) obtained through tandem mass spectrometry analysis of the excised protein band (ii) shown in Figure 4e.

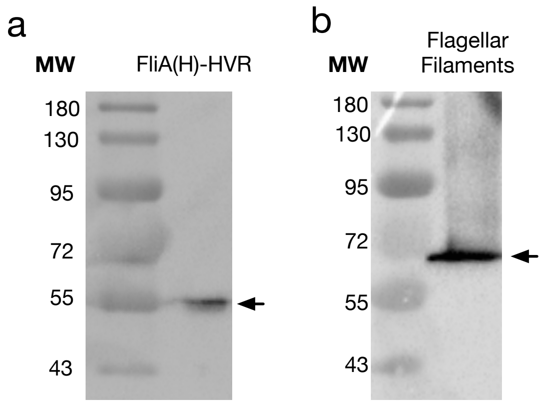

Supplementary Figure 8 | Western blot validation of the anti-FliA(H)-hypervariable region antibody. Immunoblot (10% SDS-PAGE) using (a) expressed recombinant FliA(H) hypervariable region (HVR) protease and (b) purified flagellar filaments sheared from *Clostridium haemolyticum.* The apparent molecular weights of the major bands match the predicted values [50.1 kDa for FliA(H)-HVR and 61 kDa for full length FliA(H)].

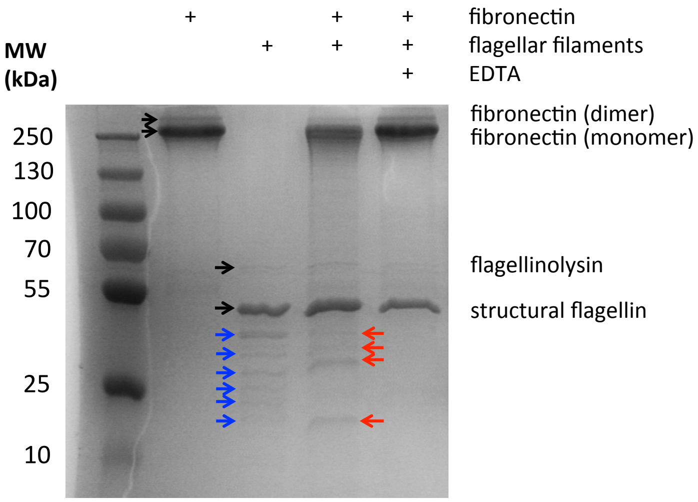

Supplementary Figure 9 | SDS PAGE (10 %) analysis of *in vitro* digestion (12 h, 37 °C) of bovine fibronectin with *C. haemolyticum* flagellar filaments containing the metalloprotease domain (flagellinolysin). Minor digestion of bovine fibronectin was apparent over 12 h generating distinct cleavage products (red arrows), including notable conversion of the 440-kDa fibronectin dimer form to the ~240 kDa monomer. The loss of the ~440 dimer indicates that a C-terminal cleavage occurs, potentially at 2403EY↓LGA2407, which matches the PICS determined cleavage specificity (Fig. 4C). This site lies N-terminal to the C-terminal interchain crosslink sites between Cys2459 and Cys2463 on fibronectin. Notably cleavage was blocked by 5 mM EDTA confirming divalent cation dependency, characteristic of metalloproteinase activity. Moreover, flagellar filament self-proteolysis of structural flagellin by flagellinolysin was also observed (blue arrows), which was not present at 0 h (see also Fig. 4e) and was lost in the presence of 5 mM EDTA. Recombinant non-polymerized flagellinolysin enzyme displayed no activity on fibronectin (not shown). This suggests that flagellinolysins in their native state, *i.e.* assembled as a component of flagellar filaments, may cleave specific host proteins and that such cleavages are potentiated by structural changes or by processivity efficiencies acquired following assembly onto a stable platform. Thus, flagellinolysin in the context of full-length flagellar filaments can cleave macromolecular protein substrates as may be present in infected animal tissues or biofilms.

Fig. 3a

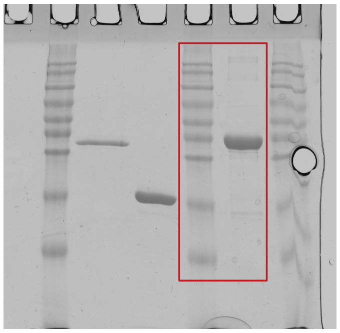

Sup. Fig. 9

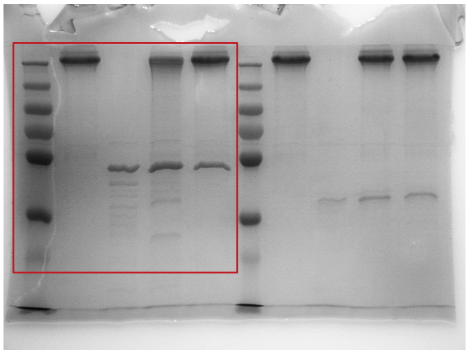

Fig. 3e

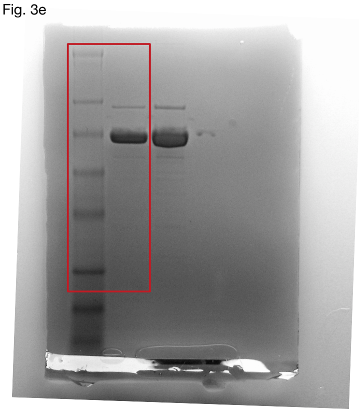

Sup. Fig. 8 (left)

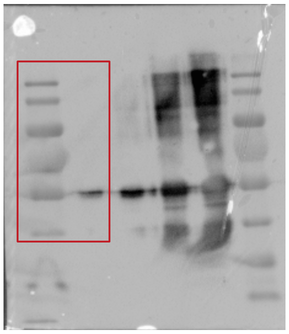

Sup. Fig. 8 (right)

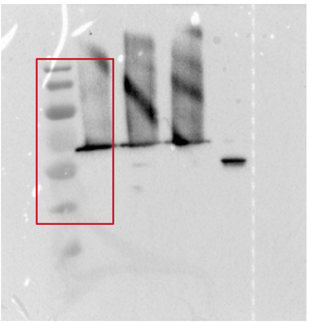

Supplementary Figure 10 | Uncropped images of gels from Figure 3 and Supplementary Figures 8 and 9.

Supplementary Table 1. Flagellin associated domain architectures and their abundance in the NCBI database as detected using the Conserved Domain Database. All flagellins analyzed contain Flagellin_N and Flagellin_C domains. Flagellin architectures with putative enzymatic domains in their hypervariable region are shaded.

# Seqs.	Taxonomy	Taxid	Accessions	Domain Architecture	Protease?
21667	Bacteria	2	pfam00669~pfam00700	Flagellin_N~Flagellin_C	
3286	cellular organisms	131567	pfam00669~pfam07196~pfam00700	Flagellin_N~Flagellin_IN~Flagellin_C	
715	Proteobacteria	1224	pfam00669~pfam08884~pfam00700	Flagellin_N~Flagellin_D3~Flagellin_C	
417	Proteobacteria	1224	pfam00669~pfam12445~pfam00700	Flagellin_N~FliC~Flagellin_C	
155	Rhizobiales	356	pfam00669~pfam07482~pfam00700	Flagellin_N~DUF1522~Flagellin_C	
67	Burkholderiaceae	119060	pfam00669~pfam12613~pfam00700	Flagellin_N~FliC_SP~Flagellin_C	
35	Bacteria	2	pfam00669~pfam01752~pfam00700	Flagellin_N~Peptidase_M9~Flagellin_C	Y
24	Bacteria	2	pfam00669~pfam11863~pfam00700	Flagellin_N~DUF3383~Flagellin_C	
21	Bacteria	2	pfam00669~cl00057~pfam00700	Flagellin_N~vWFA~Flagellin_C	
17	Acinetobacter sp. CAG:196	1262690	pfam00669~pfam05342~pfam00700	Flagellin_N~Peptidase_M26_N~Flagellin_C	Y
12	Bacteria	2	pfam00669~cl14813~pfam00700	Flagellin_N~GluZincin~Flagellin_C	Y
10	Desulfovibrionales	213115	pfam00669~cl21531~pfam00700	Flagellin_N~Sialidase~Flagellin_C	
10	Pseudomonas	286	pfam00669~pfam04004~pfam00700	Flagellin_N~Leo1~Flagellin_C	
9	Bacteria	2	pfam00669~pfam07196~cl00160~pfam00700	Flagellin_N~Flagellin_IN~LbetaH~Flagellin_C	
9	Proteobacteria	1224	pfam00669~cl14106~pfam00700	Flagellin_N~Rifin_STEVOR~Flagellin_C	
7	Bacteria	2	pfam00669~cl21456~pfam00700	Flagellin_N~Periplasmic_Binding_Protein_Type_2~Flagellin_C	
7	delta/epsilon subdivisions	68525	pfam00669~cl00160~pfam00700	Flagellin_N~LbetaH~Flagellin_C	
5	Bacteria	2	pfam00669~cl21454~pfam00700	Flagellin_N~NADB_Rossmann~Flagellin_C	
5	Enterobacteriaceae	543	pfam00669~cl12013~pfam00700	Flagellin_N~BAR~Flagellin_C	
5	Proteobacteria	1224	pfam00669~cl19731~pfam00700	Flagellin_N~SipA~Flagellin_C	
5	Proteobacteria	1224	pfam00669~cl16912~pfam00700	Flagellin_N~MDR~Flagellin_C	
4	Proteus mirabilis	584	pfam00669~cl02760~pfam00700	Flagellin_N~NEAT~Flagellin_C	
4	Bacteria	2	pfam00669~pfam12211~pfam00700	Flagellin_N~LMWSLP_N~Flagellin_C	
4	Vibrio	662	pfam00669~pfam07196~pfam14925~pfam00700	Flagellin_N~Flagellin_IN~HPHLAWLY~Flagellin_C	
4	Methylibium	316612	pfam00669~cl00261~pfam00700	Flagellin_N~PLPDE_III~Flagellin_C	
4	Butyrivibrio	830	pfam00669~pfam06156~pfam00700	Flagellin_N~DUF972~Flagellin_C	
4	Bacteria	2	pfam00669~cl15685~pfam00700	Flagellin_N~Wzt_C-like~Flagellin_C	
3	Bacteria	2	pfam00669~cl07060~pfam00700	Flagellin_N~NPCBM~Flagellin_C	
3	Bacteria	2	pfam00669~cl21487~pfam00700	Flagellin_N~OM_channels~Flagellin_C	
3	Bacteria	2	pfam00669~pfam13861~pfam00700	Flagellin_N~FLgD_tudor~Flagellin_C	
3	Gammaproteobacteria	1236	pfam00669~TIGR02597~pfam00700	Flagellin_N~TIGR02597~Flagellin_C	

3	Roseburia inulinivorans	360807	pfam00669~cl00342~pfam00700	Flagellin_N~Trp-synth-beta_II~Flagellin_C	
3	Vibrio	662	pfam00669~pfam07196~cl21456~pfam00700	Flagellin_N~Flagellin_IN~Periplasmic_Binding_Protein_Type_2~Flagellin_C	
3	Clostridium tyrobutyricum	1519	pfam00669~cl21455~pfam00700	Flagellin_N~P-loop_NTPase~Flagellin_C	
3	Carnobacterium sp. 17-4	208596	pfam00669~cl01110~pfam00700	Flagellin_N~Sdh5~Flagellin_C	
3	Bacillales	1385	pfam00669~pfam04574~pfam00700	Flagellin_N~DUF592~Flagellin_C	
3	Gluconobacter oxydans	442	pfam00669~cl11960~pfam00700	Flagellin_N~Ig~Flagellin_C	
2	Bacteria	2	pfam00669~pfam07559~pfam00700	Flagellin_N~FlaE~Flagellin_C	
2	Bacteria	2	pfam00669~cl18945~pfam00700	Flagellin_N~AAT_I~Flagellin_C	
2	Enterobacteriaceae	543	pfam00669~cl05878~pfam00700	Flagellin_N~TraK~Flagellin_C	
2	Arcobacter butzleri	28197	pfam00669~cl18951~pfam00700	Flagellin_N~Amidase~Flagellin_C	
2	Chitinibacter	230666	pfam00669~cl09928~pfam00700	Flagellin_N~Molybdopterin-Binding~Flagellin_C	
2	Bacteria	2	pfam00669~cl00192~pfam00700	Flagellin_N~ribokinase_pfkB_like~Flagellin_C	
2	Azoarcus sp. CIB	198107	pfam00669~pfam09249~pfam00700	Flagellin_N~tRNA_NucTransf2~Flagellin_C	
2	delta proteobacterium MLMS-1	262489	pfam00669~pfam07196~cl00160~cl02073~pfam00700	Flagellin_N~Flagellin_IN~LbetaH~DUF3422~Flagellin_C	
2	Bacillus alcalophilus	1445	pfam00669~pfam11589~pfam00700	Flagellin_N~DUF3244~Flagellin_C	
2	Borreliella lusitaniae	100177	pfam00669~pfam14796~pfam00700	Flagellin_N~AP3B1_C~Flagellin_C	
2	Nitrospira defluvii	330214	pfam00669~cl21542~pfam00700	Flagellin_N~EthD~Flagellin_C	
2	Firmicutes	1239	pfam00669~cl23771~pfam00700	Flagellin_N~Big_3_4~Flagellin_C	
2	Rhodopirellula	265488	pfam00669~cl10557~pfam00700	Flagellin_N~Dak1~Flagellin_C	
2	Gammaproteobacteria	1236	pfam00669~cl11964~pfam00700	Flagellin_N~CYTH-like_Pase~Flagellin_C	
2	Bacillus sp. SG-1	161544	pfam00669~cl14647~pfam00700	Flagellin_N~GH43_62_32_68~Flagellin_C	
2	Bacteria	2	pfam00669~cl14876~pfam00700	Flagellin_N~Zinc_peptidase_like~Flagellin_C	Y
2	Enterobacter cloacae	550	pfam00669~cl22495~pfam00700	Flagellin_N~Gp23~Flagellin_C	
2	Cellvibrio sp. BR	1134474	pfam00669~cl01077~pfam00700	Flagellin_N~SIMPL~Flagellin_C	
2	Idiomarina xiamenensis	1207041	pfam00669~cl00278~pfam00700	Flagellin_N~CCC1_like~Flagellin_C	
2	Rhodospirillaceae	41295	pfam00669~pfam10983~pfam00700	Flagellin_N~DUF2793~Flagellin_C	
1	Bdellovibrio bacteriovorus	959	pfam00669~cl01193~pfam00700	Flagellin_N~DUF463~Flagellin_C	
1	Paenibacillus alginolyticus	59839	pfam00669~cl21457~pfam00700	Flagellin_N~TIM_phosphate_binding~Flagellin_C	
1	Nematostella vectensis	45351	pfam00700~pfam00669	Flagellin_C~Flagellin_N	
1	Lactobacillus capillatus	480931	pfam00669~cl01389~pfam00700	Flagellin_N~Phage_sheath_1~Flagellin_C	
1	Escherichia fergusonii	564	pfam00669~cl00262~pfam00700	Flagellin_N~TroA-like~Flagellin_C	

Supplementary Table 2. Eighty-six predicted "proteolytic flagellins" identified in the NCBI database. All possess a putative gluzincin-family metallopeptidase domain in their hypervariable region.

GI	Ref	Name	Species
738243862	WP_036199097.1	hypothetical protein	Lysinibacillus sinduriensis
916691384	WP_051298475.1	hypothetical protein	Marinobacterium litorale
518372292	WP_019542499.1	hypothetical protein	Selenomonas bovis
916573195	WP_051180286.1	hypothetical protein	Selenomonas ruminantium
504238503	WP_014425605.1	putative flagellin	Selenomonas ruminantium
917028657	WP_051635369.1	hypothetical protein	Selenomonas sp. ND2010
916926872	WP_051533584.1	hypothetical protein	Anaerovibrio sp. RM50
917657302	WP_052211964.1	hypothetical protein	Anaerovibrio lipolyticus
653148213	WP_027397410.1	hypothetical protein	Anaerovibrio lipolyticus
507777340	EOS35535.1	hypothetical protein	C808_04738 Lachnospiraceae bacterium M18-1
551000896	WP_022745605.1	hypothetical protein	Dorea sp. 5-2
507824683	EOS80891.1	hypothetical protein	C817_01227 Dorea sp. 5-2
769144802	WP_044921500.1	hypothetical protein	Lachnospiraceae bacterium MA2020
910018461	WP_049973154.1	hypothetical protein	Lachnospiraceae bacterium MC2017
910018462	WP_049973155.1	hypothetical protein	Lachnospiraceae bacterium MC2017
651905107	WP_026658295.1	hypothetical protein	Butyrivibrio sp. AC2005
697062782	WP_033152383.1	hypothetical protein	Pseudobutyrivibrio ruminis
769166148	WP_044939094.1	hypothetical protein	Pseudobutyrivibrio sp. LB2011
651903221	WP_026657293.1	hypothetical protein	Butyrivibrio sp. AC2005
551017073	WP_022761310.1	flagellin	Butyrivibrio sp. AD3002
651387362	WP_026499388.1	hypothetical protein	Butyrivibrio sp. WCD2001
651394250	WP_026505968.1	hypothetical protein	Butyrivibrio sp. NC3005
551028220	WP_022772304.1	flagellin	Butyrivibrio sp. AE2015
916590076	WP_051197167.1	hypothetical protein	Butyrivibrio sp. XBB1001
490739930	WP_004602238.1	flagellin/flagellar hook associated protein	Eubacterium cellulosolvens
916920396	WP_051527108.1	hypothetical protein	Eubacterium cellulosolvens
517856800	WP_019027008.1	hypothetical protein	Colwellia piezophila
766745642	WP_044831744.1	hypothetical protein	Thalassomonas actiniarum
766760745	WP_044838529.1	hypothetical protein	Thalassomonas viridans
654652764	WP_028113749.1	hypothetical protein	Ferrimonas kyonanensis
916608721	WP_051215812.1	hypothetical protein	Ferrimonas futtsuensis
497523220	WP_009837418.1	flagellar protein	Pseudoalteromonas tunicata
654852782	WP_028305148.1	hypothetical protein	Oceanospirillum maris
654847818	WP_028300311.1	hypothetical protein	Oceanospirillum beijerinckii
518442882	WP_019613089.1	hypothetical protein	Psychromonas ossibalaenae
655481215	WP_028863050.1	hypothetical protein	Psychromonas aquimarina
917474576	WP_052080993.1	hypothetical protein	Pseudomonas sp. ML96

917193838	WP_051800550.1	hypothetical protein	Pseudomonas oleovorans
1057533868	WP_068829844.1	hypothetical protein	Pseudomonas sp. BMS12
759519778	WP_043240123.1	hypothetical protein	Pseudomonas alcaligenes
544803667	WP_021220822.1	hypothetical protein	Pseudomonas alcaligenes
919350262	WP_052807587.1	hypothetical protein	Pseudomonas sp. FeS53a
758832821	KIV71406.1	Flagellin protein FlaB	Pseudomonas sp. FeS53a
495134114	WP_007858923.1	flagellin	Acidovorax sp. CF316
950225299	WP_057269442.1	flagellin	Acidovorax sp. Root219
948088328	WP_056747048.1	flagellin	Acidovorax sp. Root568
947398799	WP_056064769.1	flagellin	Acidovorax sp. Root402
1023918667	WP_063462804.1	flagellin	Acidovorax sp. GW101-3H11
498146157	WP_010460313.1	flagellin	Acidovorax radicis
950228284	WP_057272374.1	flagellin	Acidovorax sp. Root267
950175389	WP_057223076.1	flagellin	Acidovorax sp. Root275
493342448	WP_006299301.1	flagellin	Hylemonella gracilis
737635795	WP_035605832.1	flagellin	Hylemonella gracilis
1054768843	WP_066532860.1	flagellin	Comamonas terrigena
759662642	WP_043380247.1	flagellin	Comamonas aquatica
835203907	WP_047395168.1	hypothetical protein	Chitinibacter sp. ZOR0017
654999239	WP_028448441.1	hypothetical protein	Chitinibacter tainanensis
517221226	WP_018410044.1	hypothetical protein	Methyloversatilis thermotolerans
1059956041	WP_069037803.1	hypothetical protein	Methyloversatilis sp. RAC08
1059488308	AOF81425.1	hypothetical protein BSY238_565	Methyloversatilis sp. RAC08
522143982	WP_020655191.1	hypothetical protein	Massilia niastensis
916699479	WP_051306570.1	hypothetical protein	Massilia alkalitolerans
947778910	WP_056440654.1	hypothetical protein	Massilia sp. Root335
738284338	WP_036238512.1	hypothetical protein	Massilia sp. JS1662
947464650	WP_056129008.1	MULTISPECIES: hypothetical protein	Massilia
500090247	WP_011766260.1	flagellin	Azoarcus sp. BH72
1043078958	WP_065340783.1	hypothetical protein	Azoarcus olearius
847184073	WP_047967967.1	hypothetical protein	Vogesella sp. EB
938316768	WP_054620994.1	hypothetical protein	Rhodocyclaceae bacterium Paddy-1
737307010	WP_035289894.1	hypothetical protein	Clostridium sp. KNHs214
746179135	WP_039238750.1	hypothetical protein	Clostridium novyi
746201658	WP_039258875.1	hypothetical protein	Clostridium botulinum
746169334	WP_039229452.1	hypothetical protein	Clostridium haemolyticum
**19910973**	**BAB87738.1**	**flagellin protein FliA(H)**	**Clostridium haemolyticum**
746189539	WP_039247767.1	hypothetical protein	Clostridium novyi
737261571	WP_035245515.1	hypothetical protein	Desulfobulbus mediterraneus
492846401	WP_006000355.1	flagellin-like	Desulfuromonas acetoxidans
550893434	WP_022654199.1	flagellin	Aquaspirillum serpens
1062631149	WP_069313537.1	hypothetical protein	Piscirickettsia sp. Y2

737346898	WP_035329181.1	hypothetical protein	Bacillus firmus
766666229	WP_044826104.1	hypothetical protein	Clostridium aceticum
738759210	WP_036652630.1	hypothetical protein	Paenibacillus wynnii
504121263	WP_014355249.1	flagellin	Acetobacterium woodii
503951982	WP_014185976.1	flagellin/flagellar hook associated protein	Desulfosporosinus orientis
505207458	WP_015394560.1	flagellin	Clostridium saccharoperbutylacetonicum
1011282853	WP_062200331.1	hypothetical protein	Bacillaceae bacterium mt8

Supplementary Table 3. Identified proteins and their MS/MS spectral numbers from LC-MS/MS analysis of in-gel digests (FDR ≤ 1%).

UniProt	Gene	Protein Description	Total Indep. Spectra	Percent Share of Spectrum Ids
Q8RR94 (mod)	Trx::FliA(H)-HVR	Trx::FliA(H)-HVR	11630	95.1
P17169	GLMS_ECOLI	Glutamine-fructose-6-phosphate transaminase	242	2.0
P0A6Y8	DNAK_ECOLI	Chaperone protein DnaK	131	1.1
P0A9K9	SLYD_ECOLI	FKBP-type peptidyl-prolyl cis-trans isomerase	105	0.9
P0A6T5	GCH1_ECOLI	GTP cyclohydrolase 1	32	0.3
P77398	ARNA_ECOLI	Bifunctional polymyxin resistance protein ArnA	21	0.2
P0ACJ8	CRP_ECOLI	cAMP-activated global transcriptional regulator CRP	22	0.2
P0A7M2	RL28_ECOLI	50S ribosomal protein L28	17	0.1
P76270	MSRC_ECOLI	Free methionine-R-sulfoxide reductase	12	0.1
P64588	YQJI_ECOLI	Transcriptional regulator YqjI	11	0.1
P0AFD1	NUOE_ECOLI	NADH-quinone oxidoreductase subunit E	12	0.1

Supplementary Discussion

Specificity differences between flagellinolysin and collagenase. Flagellinolysin has a deep-pocket MMP-like specificity subsite in S1', inferred from the P1' specificity and structural models, with a strong preference for leucine, isoleucine and phenylalanine. However, flagellinolysin lacks the second MMP hallmark preference for proline in P3, selecting for valine, isoleucine, and threonine instead. Nonetheless, as P3 is distant from P1' this may relax the P3 specificity to accommodate the less preferred proline in some substrates explaining the experimentally observed cleavage of QF24 (PLG↓L). Medium sized hydrophobic residues such as alanine, valine, isoleucine and proline were preferred in both libraries in P2' and P3'. An intermediate preference for polar residues such as asparagine, glutamine, and threonine was also revealed in both peptide libraries for positions P2 and P1 (Fig. 4b)—the specificity profile of which is also in agreement with our QF peptide assays, since PLG↓V was not cleaved (Fig. 4d) and valine is not preferred at P1' (Fig. 4b).
